# Evaluating the impact of chaotropic salts on protein corona formation on polyethylene glycol-*b*-polylactic acid polymersomes

**DOI:** 10.1016/j.jcis.2025.138195

**Published:** 2025-06-15

**Authors:** Owen Tabah, Daniel Nichols, Ashley Blake, Grace Witt, Chau-wen Chou, Jessica Larsen

**Affiliations:** a Department of Bioengineering, Clemson University, Clemson, SC, United States; b Department of Chemical and Biomolecular Engineering, Clemson University, Clemson, SC, United States; c Department of Chemistry, University of Georgia, Athens, GA, United States

**Keywords:** Protein corona, Polymersome, Chaotropic salts, Poly(ethylene glycol) (PEG)

## Abstract

Polymersomes (PS) are a class of hollow polymeric nanoparticle vesicles made of amphiphilic block co-polymers that self-assemble via hydrophobic interactions. One of the significant unsung challenges for their translation is the uncontrolled formation of the protein corona, which can influence PS biodistribution, cellular uptake, and immune recognition. Despite the major benefits associated with PS, no studies have yet explored engineering their protein corona. Evidence suggests that the confirmation of polyethylene glycol (PEG) chains, which can vary in response to Hofmeister series salts, can affect protein corona composition. Here, we investigated the impact of different Hofmeister series salt ions, focusing on increasing chaotropic salts [NaCl (Na^+^) < CaCl_2_ (Ca^2+^) < MgCl_2_ (Mg^2+^)] on the biomolecular identity of PEG-*b*-polylactic acid (PLA) PS after incubation in serum. We observed that the ionic environment significantly influences the protein corona formation on PEG-*b*-PLA PS. The presence of different salt ions, particularly divalent cations like calcium and magnesium, can change the size and surface chemistry of PEG-*b*-PLA PS, leading to alterations in the specific protein composition of the corona. We propose that these protein corona differences are driven by both (1) charge-based and (2) biologically driven interactions. This knowledge could be leveraged to engineer nanoparticles with tailored protein coronas. While this research focused primarily on PS made of one polymer, PEG-*b*-PLA, other polymers and polyelectrolytes in PSs need to be investigated. We’ve shown that a surface coated with low molecular weight PEG can be impacted by ions, despite not having any ionizable groups.

## GRAPHICAL ABSTRACT

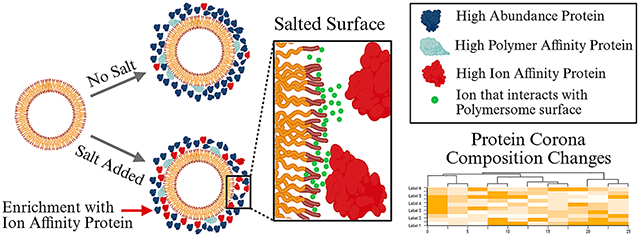

## 1. Introduction

Polymersomes (PS) are a class of hollow polymeric nanoparticle vesicles capable of delivering a diverse range of therapeutics. They have gained attention from researchers worldwide due to their ease of synthesis, versatility, tunable membrane properties, and high colloidal stability [1–3]. PSs are made of amphiphilic block co-polymers that self-assemble via hydrophobic interactions when exposed to a hydrated environment [4,5]. Their morphology resembles liposomes, consisting of an aqueous interior typically surrounded by a bilayer membrane. However, PSs have increased membrane stability, which leads to decreased diffusion of payloads out of the carrier compared to their lipid counterparts [6,7]. Choosing the correct hydrophilic and hydrophobic polymers, their degree of polymerization, as well as the ratio between them are vital to optimizing the membrane properties and morphology of PSs [8,9]. There is a wide range of polymers used in PS formulations; the most commonly used polymers for the hydrophobic block are poly (caprolactone) (PCL), poly(acrylates), and poly(lactic acid) (PLA), while the hydrophilic blocks are most commonly poly(ethylene glycol) (PEG) and poly(amino acids) [10]. In essence, the modularity and membrane properties of PS have proven them to be promising drug carriers.

As with all therapeutic carriers, PS will interact with macromolecules in a range of biological environments, specifically the bloodstream. The successful translation of PS into clinical applications hinges on their ability to navigate these biological environments and interact effectively with target cells and tissues. One of the significant unsung challenges for the successful translation of these PS is the uncontrolled formation of the protein corona [3], a dynamic layer of adsorbed proteins that forms on nanoparticles upon contact with biological fluids, effectively determining the *in vivo* fate of PS [11,12]. On the exterior of the PS membrane, both facing the surrounding environment and the vesicle core, is a dense hydrated brush of the hydrophilic component of the co-polymer, which will modulate the interactions between the PS and circulating proteins. The composition of the protein corona depends on the concentration of proteins in the fluid and the interactions between specific proteins and the PS surface. The corona composition can significantly influence nanoparticle biodistribution, cellular uptake, and immune recognition [13,14]. Some studies suggest tissue targeting is possible based on the protein corona composition [15–18]. Adding to the complexity of this phenomenon, disease states can modulate the protein composition in biological fluids and thus alter the protein corona composition on polymeric nanoparticles [19–21]. Since the outer brush layer of the PS membrane will have the most significant interactions with the proteins, it is important to choose the hydrophilic region of the co-polymer wisely for any given application. In many cases, PEG is used due to its known anti-fouling properties, increasing the circulation time of PS [22,23]. However, the formation of a protein corona on PEGylated nanoparticles is inevitable and potentially crucial for the stealth effect [24,25] Recently, researchers have even begun to explore the idea of an “engineered” protein corona, identifying various nanoparticle parameters that can aid in the formation of highly specific protein coronas after nanoparticle injection [20,26–28].

Recent evidence suggests that the confirmation of the PEG chains can affect protein corona composition and, thus, the stealth properties of PEGylated nanoparticles [29]. Several factors can affect the conformation of PEG at the nanoparticle surface. One known factor is the length and packing density of PEG chains. Research using various types of nanoparticles has demonstrated that PEG can be manipulated in molecular weight, grafting density, and confirmation to alter the surrounding protein corona [30–38]. However, this factor is controlled for with the formation of PS with hydrophilic PEG, as it is structurally comprised of a highly dense PEG layer. Additionally, PEG has been shown to act as a polyelectrolyte, meaning that there may be direct interactions between PEG chains, aqueous ions, and proteins [39–42]. Despite this fact, there is little research showing how the ionic environment around PEG affects conformation and protein adsorption on PSs. Generally, macromolecules interact with ions following the Hofmeister series, inducing either salting in or salting out effects, with chaotropic salts enabling the denaturing of proteins [43,44]. Simultaneously, when in a co-polymer, surface-grafted PEG can also vary in conformation, hydration, and elasticity in response to Hofmeister series ions [45,46]. Combining these facts, we hypothesized that we could alter protein corona composition on the surface of PEG-*b*-PLA PSs by introducing chaotropic salts, which should impact both the proteins present in serum and the PEG brush on the surface.

To explore this hypothesis, we investigated the impact of different Hofmeister series salt ions, focusing on increasing chaotropic salts [NaCl (Na^+^) < CaCl_2_ (Ca^2+^) < MgCl_2_ (Mg^2+^)] on the biomolecular identity of PEG-*b*-PLA PS after incubation in fetal bovine serum (FBS). Incubation with salts led to characteristic PS property changes, specifically concerning their surface charge. By characterizing the protein corona, we were able to identify salt-induced changes in its composition, demonstrating the potential to engineer the protein corona of PSs for the first time to our knowledge. The results presented herein demonstrate that the addition of salt ions, particularly divalent cations like Ca^2+^ and Mg^2+^, can significantly alter the protein corona composition of PEG-*b*-PLA PS. Some of these changes were found to be biologically specific, particularly regarding the adsorption of calcium-binding proteins after incubation with CaCl_2_. These findings highlight the importance of considering the ionic environment in designing and optimizing PSs as drug delivery systems. A deeper understanding of how ions modulate the protein corona on PS could pave the way for developing more effective nanotherapeutics. The addition of post-nanoparticle formation salts provides an easier alternative to engineering the protein corona than the modulation of PEG properties. It could be easier to integrate directly into the clinic with existing technologies. Subsequently, because we know that the protein corona controls the *in vivo* cellular fate, learning mechanisms to control or engineer the *in vivo* corona composition opens the doors to translating these delivery vesicles from bench to bedside.

## 2. Materials and methods

### 2.1. Materials

PEG(1000)-b-PLA(5000) was purchased from Polysciences (Warrington, Pennsylvania, USA). 300 kDa Float-A-Lyzers were purchased from Spectrum Laboratories, a Repligen company (Rancho Dominguez, California, USA). Fetal Bovine Serum was purchased from Corning (Corning, New York, USA). 4–20 % Bio-Rad Mini PROTEAN TGX, Coomassie G-250, and Precision plus protein standard were purchased from Bio-Rad (Hercules, California, USA). In-Gel Tryptic Digestion Kit (Catalog #89871) and Laemmli Buffer were purchased from Thermo Fisher Scientific (Waltham, Massachusetts, USA). All salts used, NaCl, CaCl_2_, and MgCl_2_, were purchased from Sigma Aldrich (St. Louis, Missouri, USA).

### 2.2. Methods

#### 2.2.1. PS formation using solvent injection method

PEG(1000)-*b*-PLA(5000) PSs were formed following the protocol outlined by Pierce et al. [47]. For the solvent injection, 0.4 mL of 15 mg/mL of the block co-polymer dissolved in DMSO was injected into 4 mL of water. DMSO removal was performed for 1 h using a 5 mL 300 kDa dialysis membrane. Following organic solvent removal, the PSs were divided into 4 1 mL samples.

#### 2.2.2. PS salt dialysis

Salt solutions were made with the following concentrations: NaCl: 0, 50, 100, and 200 mM; CaCl_2_: 0, 25, 50, 100 mM; MgCl_2_: 0, 25, 50. 100 mM. The molarity of the solutions was chosen to keep the concentration of chloride ions consistent. PS samples were added to their own clean 1 mL 300 kDa dialysis membrane. Loaded dialysis devices were placed in beakers containing the various salt solutions and stirred at 100 RPM overnight. PS samples were removed from the dialysis device and placed in labeled glass vials. Samples denoted as 0 mM salt underwent no additional dialysis beyond DMSO removal and were used as controls.

#### 2.2.3. Preliminary characterization

Preliminary PS characterization was completed using a Malvern Zetasizer for Dynamic Light Scattering (DLS) and Zeta Potential measurements. To obtain accurate Zeta Potential results with minimal noise, excess salt was removed from the solution by pelleting the PS samples with centrifugation (15,000 RPM for 15 min), removing the supernatant, then resuspending the PS in Milli-Q water prior to reading to limit Debye screening and probe the strength of interactions between ions and PEG [48,49]. Transmission electron microscopy (TEM) was performed using a Zeiss Em10 Transmission Electron Microscope (HT: 60 kV, Exposure time: 2.00 *sec*) after background staining with phosphotungstic acid (PTA).

#### 2.2.4. PS incubation in fetal bovine serum

Each dialyzed PS sample was transferred through a 0.22 mm PES syringe filter to an autoclaved RNA/DNA polymerase-free 1.5 mL microcentrifuge tube along with 0.5 mL FBS in a sterile environment. Samples were incubated in FBS at 37 °C for 4 h with constant shaking to mimic physiological conditions. Following incubation, PS samples were pelleted via centrifugation at 15,000 RPM for 15 min. The supernatant was removed, leaving only the pellet in the tube. Each sample was then resuspended with 1 mL of Milli-Q water. Characterization of these PS after salt dialysis and serum incubation was completed using a Malvern Zetasizer for DLS and Zeta Potential measurements.

#### 2.2.5. SDS-PAGE for total protein corona sample preparation

After incubation, PS samples were pelleted via centrifugation at 15,000 RPM for 15 min. After removing the supernatant, 100 mL of 1X Laemmli Sample Buffer was added to the pelleted PSs. PSs were resuspended in the buffer solution through pipetting and vortexing. Afterwards, samples were prepped for sodium dodecyl sulfate–polyacrylamide gel electrophoresis (SDS-PAGE) by heating at 95 degrees Celsius for 5 min.

#### 2.2.6. SDS-PAGE for protein corona characterization

SDS-PAGE was performed using a Bio-Rad Mini PROTEAN Tetra Vertical Electrophoresis Chamber. 1 Bio-Rad Mini-PROTEAN TGX gel was used for each tested salt, yielding 3 gels total. 5 mL of protein ladder were added to one well, and 10 mL of each sample were added to four subsequent wells for each salt (0 mM control and three tested concentrations). Each gel was run at 120 V for approximately 75 min. Gels were stained using Coomassie G-250 and imaged on a white background using a custom white no-filters imaging protocol.

#### 2.2.7. PS corona characterization through LC/MS

##### 2.2.7.1. In-Gel Digestion.

Following pelleting, surfactant removal, and resuspension, samples were prepared for LC/MS by running a short SDS-PAGE at 90 V for 10 min [18], with one gel for each salt and excluding the protein ladder. Gels were removed from their casings and stained using Coomassie G-250. Protein bands from each well were excised using a scalpel. In-gel digestion was carried out using a Thermo Fisher Scientific In-Gel Tryptic Digestion Kit following the manufacturer-provided protocols.

##### 2.2.7.2. Liquid Chromatography-tandem mass spectrometry (LC-MS/MS).

The LC/MS analyses were performed on a Thermo-Fisher LTQ Orbitrap Elite Mass Spectrometer coupled with a Proxeon Easy NanoLC system (Waltham, MA) located at the Proteomics and Mass Spectrometry Facility, University of Georgia.

The peptides were resuspended in 10–20 μL of 5 % acetonitrile/ 0.1 % formic acid, then loaded 0.5–1 μL into a reversed-phase ~ 15 cm long 100 μM id column (self-packed with Dr. Maisch ReproSil-pur C18AQ 120 Å 3 μM resin), then directly eluted into the mass spectrometer. Briefly, the two-buffer gradient elution (0.1 % formic acid as buffer A and 99.9 % acetonitrile with 0.0.1 % formic acid as buffer B) starts with 5 % B, holds at 5 %B for 2 min, then increases to 25 % B in 55 min and to 50 %B in 10 min, to 95 % B in 5 min.

The data-dependent acquisition (DDA) method was used to acquire mass spectrometry data. A survey MS scan was acquired first, and then the top 12 ions in the MS scan were selected for following collision-induced diffusion MS/MS analyses. Both MS and MS/MS scans were acquired by Orbitrap at the resolutions of 120,000 and 15,000, respectively.

Data were acquired using Thermo Xcalibur software (version 3.0). Protein identification and label-free quantification were performed using Thermo Proteome Discoverer (version 3.0, Thermo Scientific) with Mascot (Matrix Science, London, UK) against Bos taurus of Swiss-Prot database (UniProt.org).

The data search parameters include:

- Mass tolerance: 10 ppm for peptide ions, and 0.02 Da for the MS/MS fragment ions
- Fixed modification: carbamidomethyl on cysteine
- Variable Modification: oxidation on methionine
- Trypsin missed cleavage: 2

The search results were validated with a reversed decoy database by Percolator [50].

The label-free quantification workflow aligns the retention times of peptides within 10 min. The peptide abundance is based on the peak area at the minimum signal-to-noise ratio (S/N) of at least 5. Here, the shared and unique peptides are counted into the most confident protein matches. The normalization of the abundances of the 12 samples is based on their total peptide amounts, and the protein abundance is calculated using the summed abundance of peptide matches.

The final protein matches are filtered by a minimum two unique peptide matches, False Discovery Rate (FDR) at 5 % and Mascot score of 20 in any of the samples in quantitative analysis.

#### 2.2.8. Jaccard Similarity

Jaccard similarities were calculated by comparing the identified proteins in each salted group using the following equation:

(1) $$J\left(A,B\right)=\frac{\left|A\cap B\right|}{\left|A\cup B\right|}$$

Where A and B represented the comparative sets, i.e. each salt and concentration was compared against each other salt and concentration.

#### 2.2.9. Statistical Analysis

All DLS data, including Z-average diameters, polydispersity index (PDI), zeta potential, and conductivity were compared within each salt group using a Brown-Forsythe and Welch One Way ANOVA test with a Dunnett’s T3 multiple comparison test. Assessments were performed in GraphPad Prism 10.4.1.

## 3. Results and discussion

### 3.1. Effect of chaotropic salts on PS properties

PEG-*b*-PLA PS were characterized both before (0 mM) and after adding three chaotropic salts: NaCl, CaCl_2_, and MgCl_2_. [51–54] All salt concentrations explored were matched for valency to ensure the same ion concentration was in the solution. This allowed us to compare findings across consistent osmotic pressure (Table 1). TEM images confirmed the formation of the expected PS membrane pre-salt incubation (Fig. S1), which matches our previous findings [51–54] As shown, the average hydrodynamic diameter of all samples was ~ 170 nm with an average monodisperse PDI of ~ 0.04 regardless of the salt added post-formation. Little to no change in size or PDI was observed when adding any chaotropic salt. Furthermore, all our samples fall within a generally acceptable range for diverse drug delivery applications and avoidance of biological barriers [55].

**Fig. 1.**
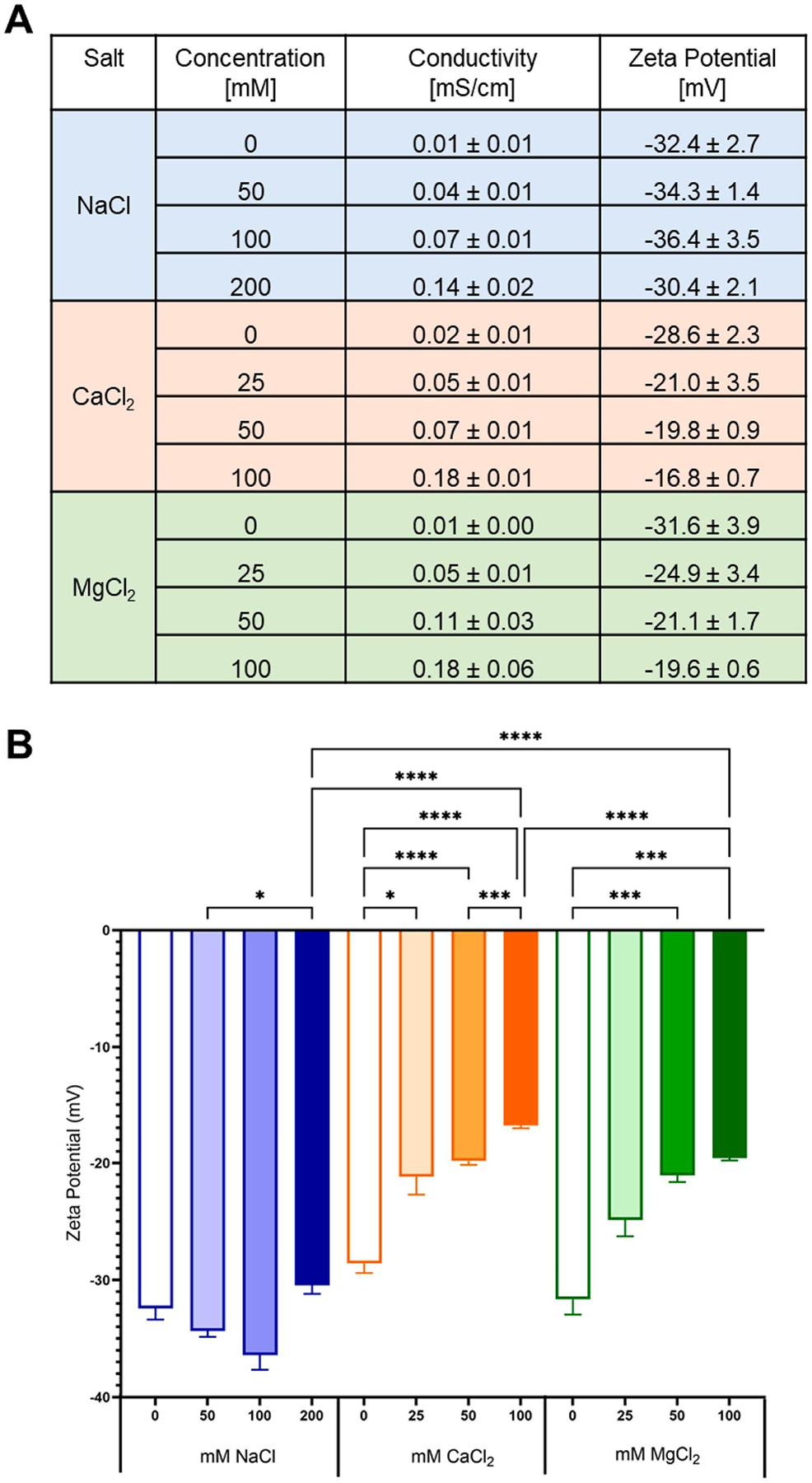
Surface charges of PEG-*b*-PLA polymersomes after dialysis against chaotropic salts at varying concentrations. A. Conductivity (mS/cm) and Zeta Potential (mV) of polymersomes after dialysis against salts is presented as mean ± standard deviation. Conductivity data suggests an increase in conductivity as the concentration of each salt increases, although not statistically significant. Zeta potential measurements provide more details into this salt affinity. B. Zeta potential is plotted across all concentrations of salt explored. Statistical increases in zeta potential with increasing salt concentration are observed after incubation with CaCl_2_ and MgCl_2_. * p < 0.05, ** p < 0.005, *** p < 0.0005, **** p < 0.0001 (n = 3).

Despite the lack of statistical significance, findings do suggest that PS average diameter modestly decreases with the addition of salt, with average diameters changing by ~ 8 nm, ~4 nm, and ~ 5 nm after the addition of the highest concentrations of NaCl, CaCl_2_, and MgCl_2_, respectively. Notably, standard deviations in diameter remained consistent with each internal pre-salt (0 mM) PS sample. This slight decrease in diameter could be due to osmotic shrinking, with the addition of salt causing the PSs to condense [8]. This subtle shift in size could also be attributed to changes at the surface due to the low permeability of PS membranes [7]. Simulation studies have shown that PEG can act as a polyelectrolyte in an aqueous environment [39], conformationally changing in response to salt ions [56]. Specifically, high levels of negatively charged salting-out ions, such as chloride, can collapse PEG brushes to a condensed brush regime [45]. Other research has reported a similar phenomenon and highlighted PEG’s ability to act as a weak polyelectrolyte, adopting different conformations in response to changes in the ionic environment [57]. Kou et. al identified that positively charged chaotropic ions dehydrate and collapse the structure of negatively charged polymer brushes (poly(3-sulfopropyl methacrylate potassium), with the impact being non-ion specific [56]. As seen in Fig. 1A, PEG-*b*-PLA PSs are negatively charged in an aqueous environment. Therefore, we can hypothesize that the chaotropic salts would impact the PEG conformation in our PS system and decrease the overall diameter. However, future studies need to be conducted to confirm this. Specifically, Small-Angle X-ray Scattering or static light scattering could determine the radius of gyration (Rg) of the PS, which will help estimate the conformation of PEG based on the ratio of Rg to hydrodynamic radius [58].

To accurately assess the impact of different salt ions on PS surface charge, or zeta-potential, excess ions in solution were removed by centrifugation, pelleting, and resuspension in Milli-Q water. The effectiveness of this washing step was confirmed by monitoring the conductivity of the resulting solutions. Notably, the measured conductivities (Fig. 1A) were consistent across osmotic pressure-matched solutions (e.g. 50 nM NaCl, 25 nM CaCl_2_ or MgCl_2_) [59]. Conductivity increased slightly with all salts and rising salt concentrations, possibly due to PEG-bound ions. Fig. 1B demonstrates that although conductivities were similar, the zeta potential values were significantly different, also suggesting that ions were PEG-bound. The addition of monovalent Na^+^ elicited minimal changes in zeta potential, with only a slight increase in surface charge observed when comparing the 50 mM and 200 mM NaCl incubated samples. Conversely, the divalent cations Ca^2+^ and Mg^2+^ induced more substantial changes. As the concentration of these divalent cations increased, the zeta potential of the PS shifted towards more positive values. Each incremental addition of divalent salt led to corresponding increases in measured zeta potential, with a maximum change of + 12 mV observed after both MgCl_2_ and CaCl_2_. This observation suggests preferential adsorption of divalent cations onto the nanoparticle surface compared to monovalent sodium ions. Consequently, the stability of the nanoparticles may decrease due to the zeta potential neutralization, yet no aggregation was observed during this study.

Our reports are not the first to show an association between cations and a PEG-coated surface. The oxygen atoms within the PEG chains give it a partial negative charge, allowing for interactions with cations. Specifically, Bhattacharjee, et al. identified that chaotropic salts can permeate into the PEG layer of a micelle made from block co-polymer PEG-*b*-poly(propylene glycol)-*b*-PEG and interact with the hydrophobic layer in aqueous solution, leading to greater changes in polymer properties than the addition of kosmotropic salts [60] Our observed reduction in the magnitude of zeta potential is consistent with literature that shows divalent cations Ca^2+^ and Mg^2+^ preferentially adsorb to PEGylated surfaces [61]. While we observed little change associated with adding Na^+^, another study found that even the addition of monovalent ions can impact PEG’s interactions with water [46]. However, this study used much higher salt concentrations of 400 mM and didn’t wash their PEGylated surfaces after exposing them to ionic solutions. Taken together, these findings suggest that PEG can interact with both monovalent and divalent cations, but the attraction of divalent cations is stronger and more pronounced.

### 3.2. Effects of added salt on protein corona composition

To investigate the influence of salt ions on the interaction of PEG-*b*-PLA PS with biological fluids, we examined the protein corona formed on PS in the presence and absence of NaCl, CaCl_2_, and MgCl_2_. The protein corona, a dynamic layer of adsorbed proteins that forms on nanoparticles upon contact with biological fluids, is known to play a critical role in determining nanoparticle behavior in vivo, including their pharmacokinetics and biodistribution [13]. Modulating the surface chemistry of nanoparticles influences the protein corona composition, thereby altering nanoparticle fate and function [62]. Table 2 highlights the PS physicochemical changes in each sample after incubation in serum. Notably, all supramolecular aggregates of salt-incubated PS and serum proteins have higher PDI and decreased magnitude in zeta potential than their unsalted counterparts, likely due to protein binding. However, changes to Z-average diameter vary across salts. Post-incubation with NaCl (Fig. S2A) and MgCl_2_ (Fig. S2C), size distributions vary dramatically, with the appearance of multiple peaks. This suggests that proteins were weakly bound to PEG and aggregating in solution. In contrast, post-incubation with CaCl_2,_ PS size distributions are very uniform (Fig. S2B), which suggests a strong interaction between proteins and PEG. We hypothesize that Ca^2+^ ions facilitate the formation of a hard protein corona, while Na^+^ and Mg^2+^ form softer protein coronas [63].

**Fig. 2.**
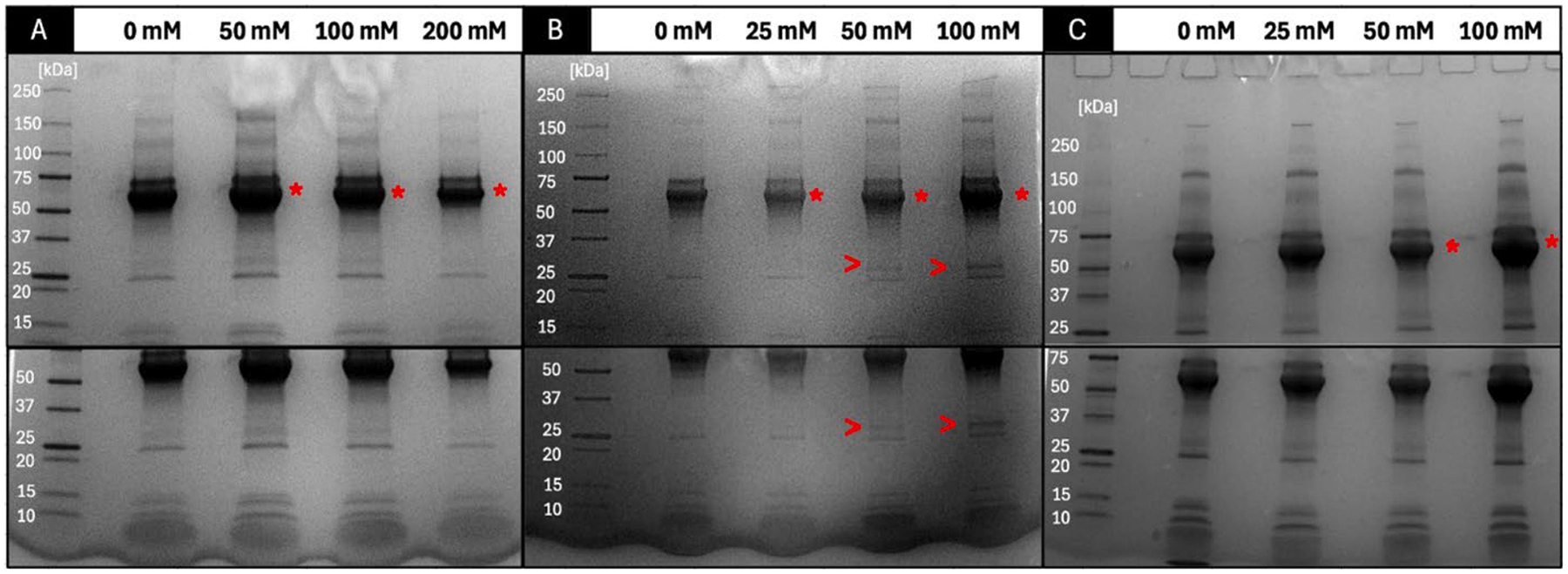
SDS-PAGE gel images of A) NaCl sample set, B) CaCl_2_ sample set, and C) MgCl_2_ sample set. Asterisks (*) are provided to highlight changes in total protein abundance seen within the darkest region and arrows (>) point to new band formation. The bottom image is the same gel, but expanded area to show the lower MW proteins. The ladder values are given in kDa. Representative images from n = 4.

SDS-PAGE analysis was performed on PS incubated with FBS, which were subsequently pelleted to remove excess proteins in the solution. The resulting gels (Fig. 2) revealed changes in protein corona profiles between salted and unsalted PS. Although the gels exhibited limited resolution in the 50–75 kDa range, potentially due to the overwhelming abundance of albumin, the most prevalent protein in serum, or differences in total protein recovered, the band intensity in this region did change (asterisks in Fig. 2). As each sample went through the same experimental steps prior to running SDS-PAGE, a decrease in protein abundance could be attributed to changes in PEG brush conformation, as highlighted above [29]. Ideally, visualizing differences in specific protein banding could provide evidence of surface chemistry change. Crucially, a novel protein band consistently appeared in the presence of CaCl_2_, not readily seen in other samples (arrows in Fig. 2B). This band’s intensity increased with rising CaCl_2_ concentration from 50 to 100 mM, demonstrating a potential correlation between Ca^2+^ ion concentration and protein binding. This observation provides strong evidence of protein corona modulation and alteration of nanoparticle surface chemistry, specifically induced by Ca^2+^ ions. These observations were confirmed by LC-MS analysis.

**Fig. 3.**
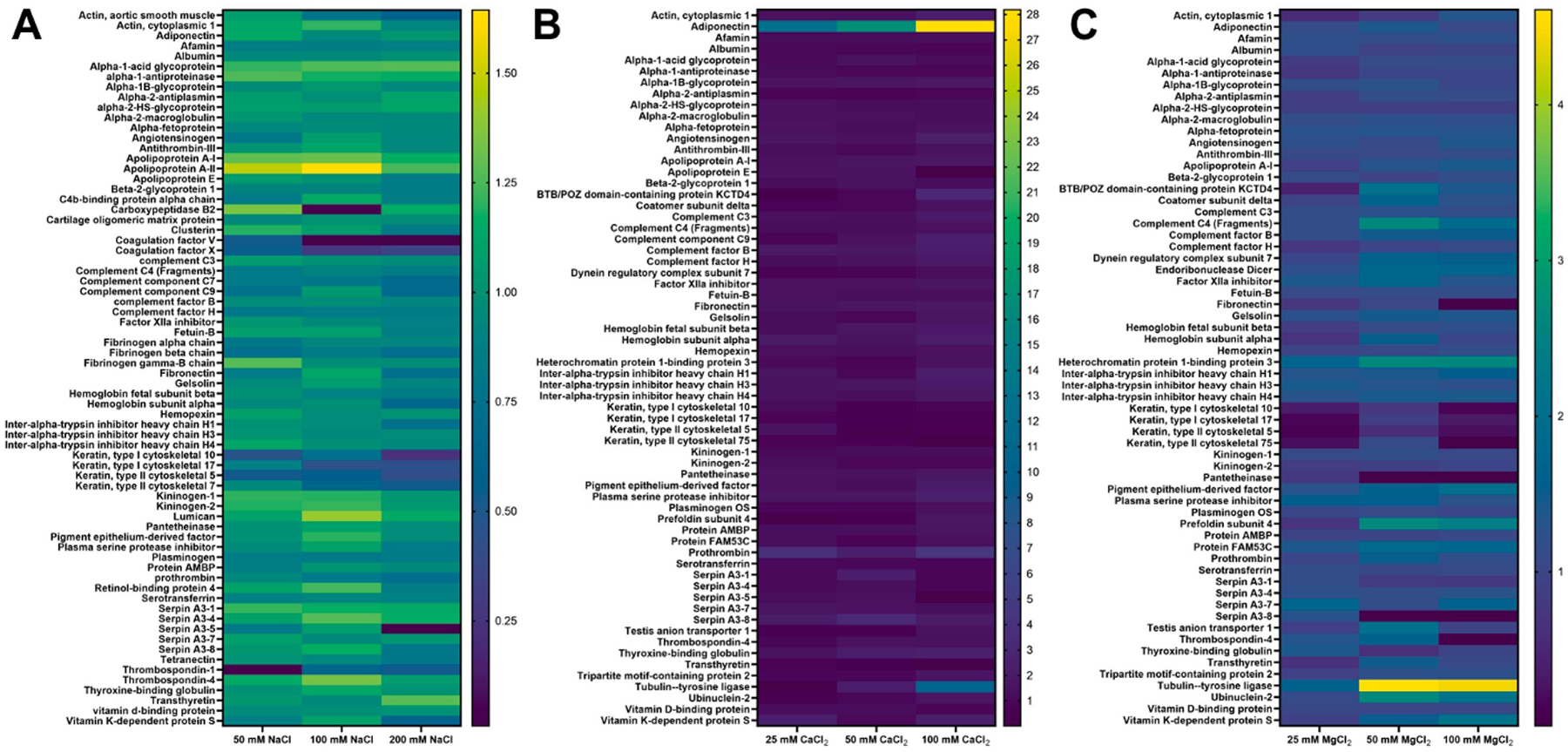
Heat maps of relative protein abundance in the protein corona detected by LC-MS/MS as compared to 0 mM controls after incubation with (A) NaCl, (B) CaCl_2_, and (C) MgCl_2_. Scale Bars: A. 0 to 1.75 fold, B. 0 to 28 fold, C. 0 to 5 fold. Heat maps are also presented as whole page images with NaCl in Fig. S3, CaCl_2_ in Fig. S4, and MgCl_2_ in Fig. S5.

To directly compare the effects of different salts, protein abundance data from salted samples were normalized to their corresponding unsalted counterparts. Heatmaps generated from this normalized data (Fig. 3) revealed that NaCl had the least impact on protein corona composition (Fig. 3A, Fig. S3). The most notable change in the NaCl group was a 1.65-fold increase in apolipoprotein A-II after high salt concentration incubation of 100 mM. These findings were confirmed by a lack of change in the top 20 most abundant proteins (Table S1). In contrast, the 50 mM MgCl_2_ sample (with equivalent chloride ion concentration to 100 mM NaCl) showed a much more dramatic ~ 5-fold increase in tubulin-tyrosine ligase compared to the unsalted control (Fig. 3C, Fig. S5). Of note is that tubulin tyrosine ligase contains magnesium binding sites and must bind to magnesium to function as an enzyme [64]. Although being upregulated, this protein did not make it into the top 20 most abundant proteins (Table S3). However, angiotensinogen was identified as a unique, abundant protein after PEG-*b*-PLA incubation in two of the three MgCl_2_ concentrations. Although it is unclear whether angiotensinogen binds directly with Mg^2+^, there is a proposed link between Mg^2+^ concentration and the renin-angiotensin system in which angiotensinogen is involved [65]. Other proteins in the MgCl_2_ group also exhibited 3-fold changes, both increases and decreases, suggesting Mg^2+^ adsorption onto the nanoparticle surface, corroborating our zeta potential findings.

Most strikingly, the CaCl_2_ group showed significant enrichment of adiponectin, a protein not prominently detected in other samples (Fig. 3B, Fig. S4) with up to 28-fold increases. This enrichment was robust enough to propel adiponectin into the corona’s top 20 most abundant proteins (Table S2). Moreover, the appearance of a new band on the SDS-PAGE gel at ~ 25 kDa (Fig. 2B), corresponding to adiponectin’s molecular weight, further supports this finding. This qualitative and quantitative evidence strongly suggests selective recruitment of adiponectin due to Ca^2+^ adsorption onto the PS, causing the changes in zeta potential observed. This is further supported by the studied interactions between calcium and adiponectin, which suggest that oligomerization of high molecular weight adiponectin is calcium-dependent and identifies a calcium-binding motif in the amino acid sequence of adiponectin [66]. This could impact PS biotransport, as extracellular vesicles with adiponectin-enriched surfaces were able to interact with adiponectin receptors on hepatocytes, with a biodistribution favoring the liver and pancreas [67]. On top of this increase in adiponectin, a 10-fold increase of tubulin-tyrosine ligase and a 4-fold increase of prothrombin was observed after incubation in 100 mM CaCl_2_. While tubulin-tyrosine ligase is not known to have a calcium-binding site, it does present a magnesium binding site. It has been shown that calcium and magnesium are competitive ligands for the same binding sites within the body [68]. Similar to MgCl_2_ incubations, CaCl_2_ incubations led to the presence of angiotensinogen in the top 20 most abundant proteins. Similarly, Ca^2+^ plays a role in the renin-angiotensin system, although it is unclear if angiotensinogen directly interacts with these ions [69].

**Fig. 4.**
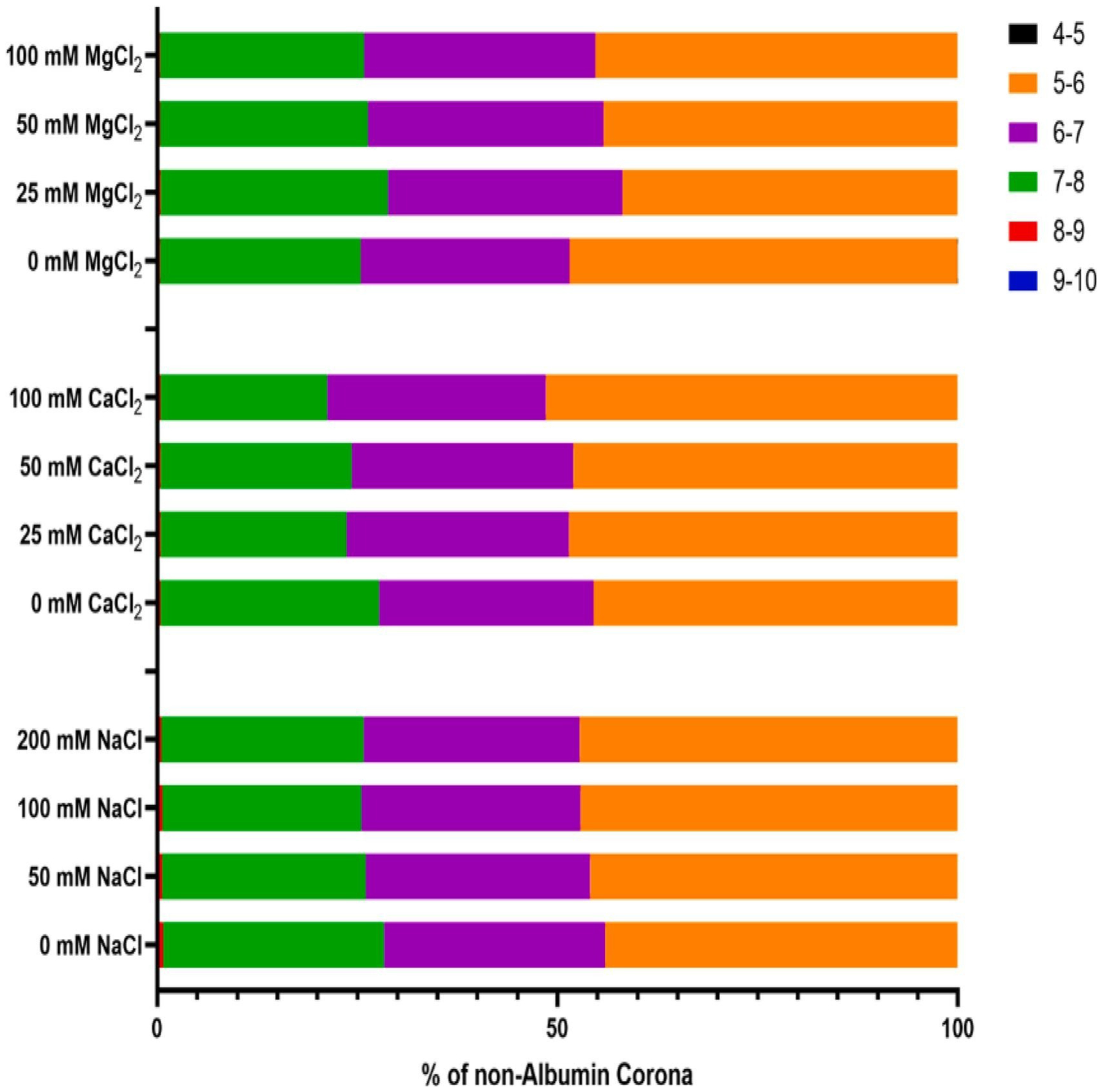
Protein corona on PEG-*b*-PLA polymersomes after incubation with chaotropic salts organized by isoelectric point (pI). The presented figure excludes albumin due to its high abundance making changes in composition hard to visualize.

To better understand the impact of chaotropic salt ions on the protein corona, the LC-MS-identified proteins were grouped by both isoelectric point (pI) and biological function. The pI of the protein corona varied little across all salt concentrations explored (Fig. S6), likely due to the high abundance of albumin, which has a pI in the 6–7 range. Once albumin is removed, differences become more obvious (Fig. 4). By comparing the 0 mM sample in each set to the most concentrated salt sample across the non-albumin protein corona, incubation with NaCl led to very modest protein corona pI changes. This matches all other data collected using NaCl (Figs. 1, 2, 3, Table S1), which indicates that the presence of Na^+^ leads to minimal changes in PEG-*b*-PLA surface properties and minimal changes in protein corona composition. Incubation with increasing concentrations of both CaCl_2_ and MgCl_2_ led to a decreased amount of positively charged (pI > 7) proteins in the corona, visualized by less red (pI 8–9) and slightly less green (pI 7–8) in Fig. 4, and an increased amount of negatively charged proteins (pI < 7) in the corona, visualized by increased purple (pI 6–7) and orange (pI 5–6) in Fig. 4. This, again, points to the surface adsorption of Ca^2+^ and Mg^2+^ ions, as confirmed through zeta potential assessments (Fig. 1). One potential mechanism that could lead to the enrichment of specific, negatively charged proteins after incubation with divalent cations is a double-chelating mechanism proposed by Wu et al. After incubation of silica nanoparticles with physiologically relevant concentrations of CaCl_2_, it was proposed that Ca^2+^ chelates the oxygen on the surface of silica nanoparticles, then bovine serum albumin (BSA), the explored protein of interest, chelates the Ca^2+^ remaining on the nanoparticle surface leading to BSA binding [70]. Similar behavior has been observed with surface-modified PEGylated gold nanoparticles, where the most significant change in protein corona with regards to pI occurred with the highest surface charge [17]. Notably, surface charge seems to have a greater impact on the protein corona pI than other factors like mechanical stiffness [71].

**Fig. 5.**
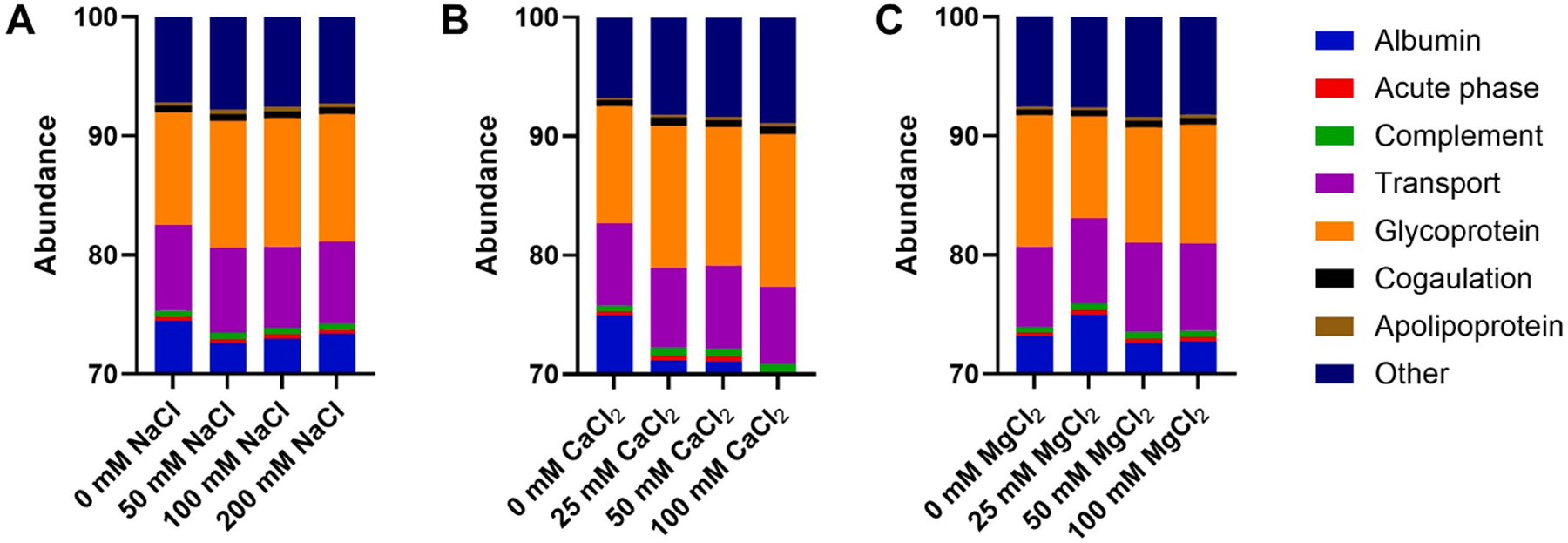
Protein corona characterized by protein function for PEG-*b*-PLA PSs incubated (A) NaCl, (B) CaCl_2_, and (C) MgCl_2_. This figure includes only 30 % of the protein corona, as > 70 % was made up of albumin. Full abundance based on protein function can be observed in Fig. S6.

Proteins were also classified according to their biological function (Fig. 5A–C). Albumin dominated all coronas, comprising 70–75 % of the total protein abundance (Fig. S7). However, CaCl_2_ exerted the most significant impact on protein corona composition with regards to their function, increasing the relative abundance of glycoproteins by ~ 3 % and other proteins by ~ 2 % while decreasing albumin abundance by ~ 5 %. NaCl had little effect on protein corona with regards to protein function, while MgCl_2_ led to only minor changes after incubation with 25 mM, which led to slight increases in albumin and decreased glycoproteins. Notably, glycoproteins are calcium-binding proteins [72–74], so if Ca^2+^ is binding or adsorbing to the PEG surface of PSs, as suggested by changing surface charges (Fig. 1), these proteins may be interacting with this surface localized calcium, thus altering the protein corona.

**Fig. 6.**
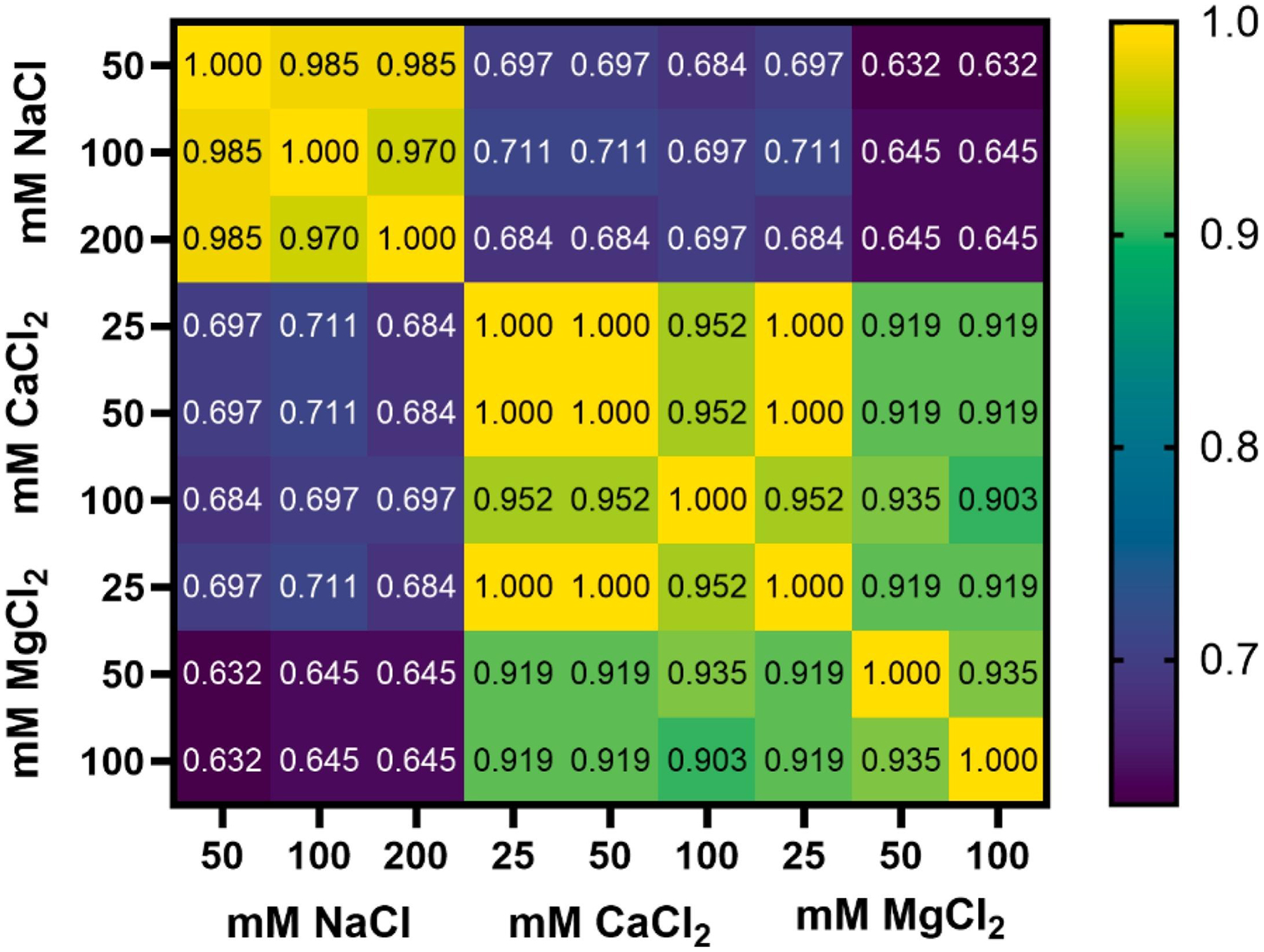
Jaccard Similarity Matrix comparing the Differences in Protein Corona Components on PEG-*b*-PLA PSs after incubation with NaCl, CaCl_2_, or MgCl_2_.

As a summary, the proteins identified by LC-MS/MS in each PEG-*b*-PLA PS protein corona were compared across all explored chaotropic ions and concentrations by calculating Jaccard similarities in the detected proteins, without accounting for their relative abundances (Fig. 6). This matrix makes it clear that the protein corona components are similar after incubation with divalent cations Ca^2+^ and Mg^2+^, which both deviate from the protein corona after incubation with monovalent cation Na^+^. Wilson and team saw similar results when studying the impact of Hofmeister series salts on PS shape modulation. Due to their higher charge-ionic radius ratio, they observed that the divalent ions bind more strongly to PEG than the monovalent ions [75]. This matches what was observed with the PEG-*b*-PLA PSs explored here. Divalent cations led to the greatest changes in surface charge (Fig. 1) and protein corona composition compared to monovalent Na^+^, which contributes to their similarities. Notably, they also identified that Ca^2+^ initiated shape changes of PEG-based PSs more strongly than Mg^2+^, which is the opposite of the standard Hofmeister series [75]. This could be contributing to some of the differences we are seeing between Ca^2+^ and Mg^2+^-induced protein coronas.

When looking at the Jaccard similarities between divalent cations, deviations between the two increase as concentrations increase. This points to a secondary mechanism likely impacting the final PEG-*b*-PLA PS protein coronas beyond charge-based interactions. Calcium-binding proteins are abundant in serum and in the bloodstream as discussed above. CaCl_2_ incubation led to the upregulation of these types of proteins, like adiponectin and other glycoproteins, within the formed protein coronas.

## 4. Conclusions

Herein, we have provided guiding principles for engineering the protein corona on PSs for the first time. Ultimately, our findings emphasize the importance of considering the ionic environment when designing and optimizing PS systems for drug delivery, as this can greatly impact their final protein corona. This protein corona is known to impact the biologic fate and tissue tropism of the PSs. We have demonstrated that the ionic environment significantly influences the protein corona formation on PEG-*b*-PLA PS. Specifically, divalent cations had a greater impact on protein corona composition than monovalent cations, while incubation of PEG-*b*-PLA PS with calcium impacted the overall protein corona composition the most. We propose that these protein corona differences are driven by both (1) charge-based interactions and (2) biologically driven interactions. Surface-associated Ca^2+^ and Mg^2+^ ions had large impact on PEG-*b*-PLA PS zeta potential, causing changes of about 60 % compared to the modest ~ 6 % change observed after incubation with Na^+^ ions (Fig. 1). These associated divalent ions, in turn, led to the greatest differences in protein corona on PSs (Fig. 3B,C), indicating that zeta-potential influenced the protein corona composition. This is further evidenced by organizing the protein corona composition based on protein pI (Fig. 4), which points to charge-based interactions. However, the impact of zeta potential alone does not describe the observed key differences between Ca^2+^ and Mg^2+^. The recruitment of calcium-binding proteins after incubation with Ca^2+^ (Fig. 2B, Fig. 3, Fig. 5B) suggests biologically specific interactions at play during protein corona formation. [46] This knowledge could be leveraged to engineer nanoparticles with tailored protein coronas. We’ve shown that a surface coated with low molecular weight PEG can be impacted by ions, despite not having any ionizable groups. Therefore, using known anti-fouling zwitterionic polymers, such as 2 methacryloyloxyethylphosphorylcholine (MPC), with this same salt dialysis technique may be able to achieve even more specific protein corona profiles or enhanced anti-fouling properties [76,77]. Prior to assessment of salt incubated PSs and their in vivo tropism, it will be important to fully characterize their performance in human serum, as it has different proteins and protein isoforms that can alter PS physicochemical properties. Investigating the *in vivo* impact of these ion-induced protein corona changes will be crucial for translating these findings into clinically relevant nanomedicine platforms, leading to improved investigation of protein coronas and their impact on the pharmacokinetics and biodistribution of polymeric nanoparticles.

## Supplementary Material

Supplementary Material

## Figures and Tables

**Table 1:** Dynamic Light Scattering (DLS) data from all samples after dialysis against salt. Statistical analysis found no differences in size or PDI within each group regardless of salt concentration. Data presented as mean±standard deviation. (n=3).

Salt	Concentration [mM]	Z-average Diameter [nm]	PDI [-]
NaCl	0	171.5±13.7	0.049±0.017
	50	165.1±13.0	0.043±0.018
	100	164.0±12.9	0.056±0.028
	200	163.3±13.3	0.044±0.020
CaCl_2_	0	178.8±6.6	0.035±0.023
	25	177.3±7.2	0.031±0.018
	50	175.8±7.7	0.040±0.014
	100	174.5±7.1	0.046±0.020
MgCl_2_	0	171.9±10.2	0.046±0.016
	25	172.6±10.1	0.034±0.014
	50	169.5±10.1	0.040±0.026
	100	165.6±11.1	0.048±0.021

**Table 2:** Dynamic Light Scattering (DLS) data from all samples post salt incubation after dialysis against serum. Data presented as mean±standard deviation. (n=4). Overall size distributions presented in Fig. S2.

		Post Serum Incubation		
Salt	Concentration [mM]	Z-average Diameter [nm]	PDI [-]	Zeta Potential [mV]
NaCl	**0**	153.6±2.8	0.037±0.025	−33.1±1.9
	**50**	119.3±25.4	0.61±0.33	−25.8±3.1
	**100**	102.6±15.4	0.75±0.22	−25.7±1.6
	**200**	299.4±149.0	0.59±0.13	−22.5±0.5
CaCl_2_	**0**	162.0±13.1	0.030±0.014	−33.7±2.8
	**25**	104.7±4.2	0.11±0.016	−18.3±1.0
	**50**	98.24±1.9	0.14±0.031	−14.9±1.0
	**100**	93.1±5.7	0.20±0.020	−13.8±2.6
MgCl_2_	**0**	158.4±10.2	0.041±0.029	−34.8±2.6
	**25**	101.7±31.1	0.68±0.053	−15.9±4.6
	**50**	90.3±55.1	0.74±0.16	−14.7±2.9
	**100**	189.3±86.8	0.72±0.24	−12.7±1.6

## Data Availability

Data will be made available on request.

## Appendix A. Supplementary data

Supplementary data to this article can be found online at https://doi.org/10.1016/j.jcis.2025.138195.
