# Supplementary material for: Evaluating the impact of chaotropic salts on protein corona formation on polyethylene glycol-b-polylactic acid polymersomes
Source: J Colloid Interface Sci. Author manuscript; Available in PMC 2025 Dec 1. (PMC12573076; doi:10.1016/j.jcis.2025.138195)
Supplement: Supplementary Material [file NIHMS2119014-supplement-Supplementary_Material.docx]

**Supporting Information**

**Evaluating the Impact of Chaotropic Salts on Protein Corona Formation on Polyethylene Glycol-b-Polylactic acid Polymersomes**

Owen Tabah ^a^, Daniel Nichols ^b^, Ashley Blake ^b^ , Grace Witt ^a^, Chau-wen Chou^c^, Jessica Larsen^a,b, *^

^a^ Department of Bioengineering, Clemson University, Clemson, South Carolina

^b^ Department of Chemical and Biomolecular Engineering, Clemson University, Clemson, South Carolina

^c^ Department of Chemistry, University of Georgia, Athens, Georgia

** Corresponding Author: 130 Earle Hall, Clemson, SC 29634,* [*larsenj@clemson.edu*](mailto:larsenj@clemson.edu)

*
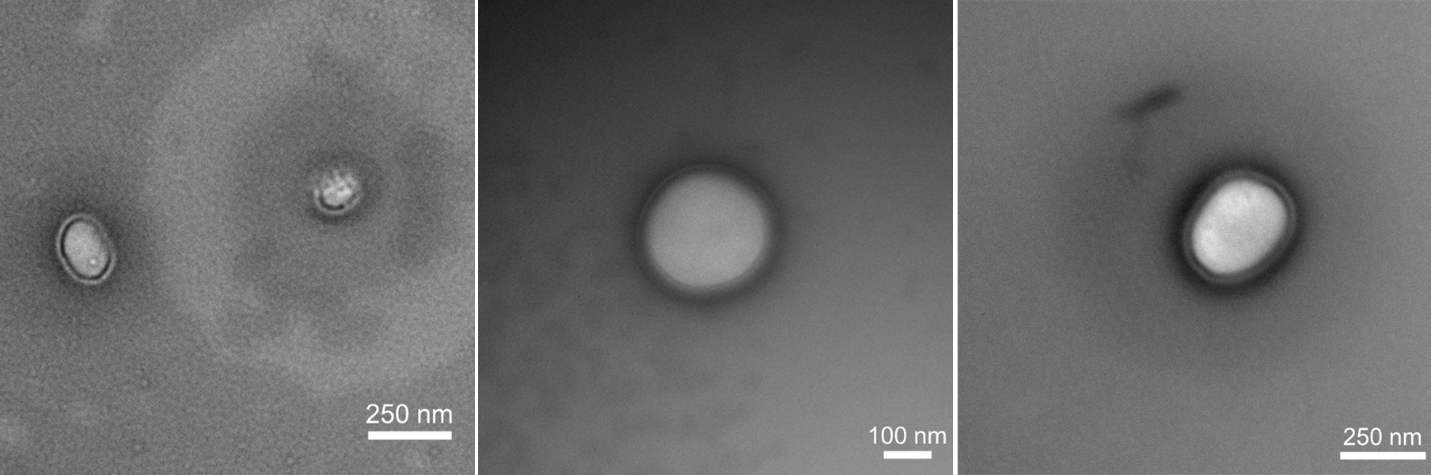
*

**Figure S1. TEM images of PEG-b-PLA polymersomes obtained using a Zeiss EM10 TEM and PTA background stain.** Membranes are clearly visible and polymersomes have diameters around the expected sizes, as calculated via DLS (Table 1).


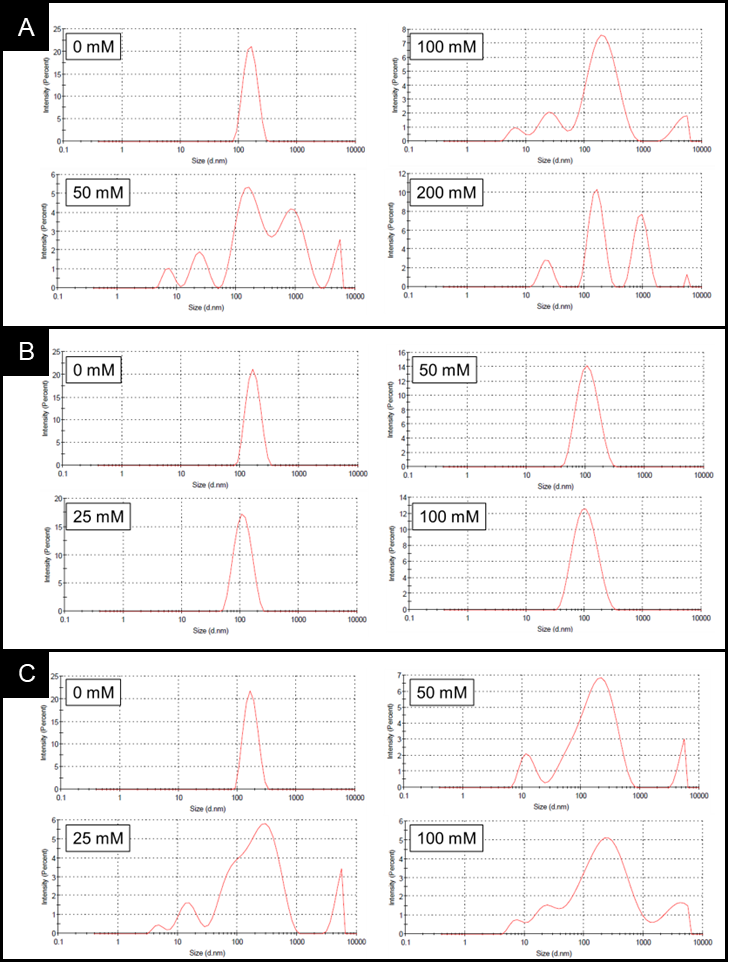


**Figure S2. Dynamic Light Scattering Intensity-Weighted Size Distributions Post Serum incubation for PEG-PLA PS + Salt** (A) NaCl, (B) CaCl_2_, (C) MgCl_2_. (n=4, but only one representative measurement shown here).

**Table S1.Top 20 Most Abundant Proteins, as detected by LC-MS, after incubation with NaCl at various concentrations.** Gray text indicates a change in position from the 0 mM NaCl control.

**Table S2. Top 20 Most Abundant Proteins, as detected by LC-MS, after incubation with CaCl_2_ at various concentrations.** Gray text indicates a change in position from the 0 mM CaCl_2_ control. Red text indicates a novel protein compared to the 0 mM CaCl_2_ control.

**Table S3.** **Top 20 Most Abundant Proteins, as detected by LC-MS, after incubation with MgCl_2_ at various concentrations.** Gray text indicates a change in position from the 0 mM CaCl_2_ control. Red text indicates a novel protein compared to the 0 mM MgCl_2_ control.


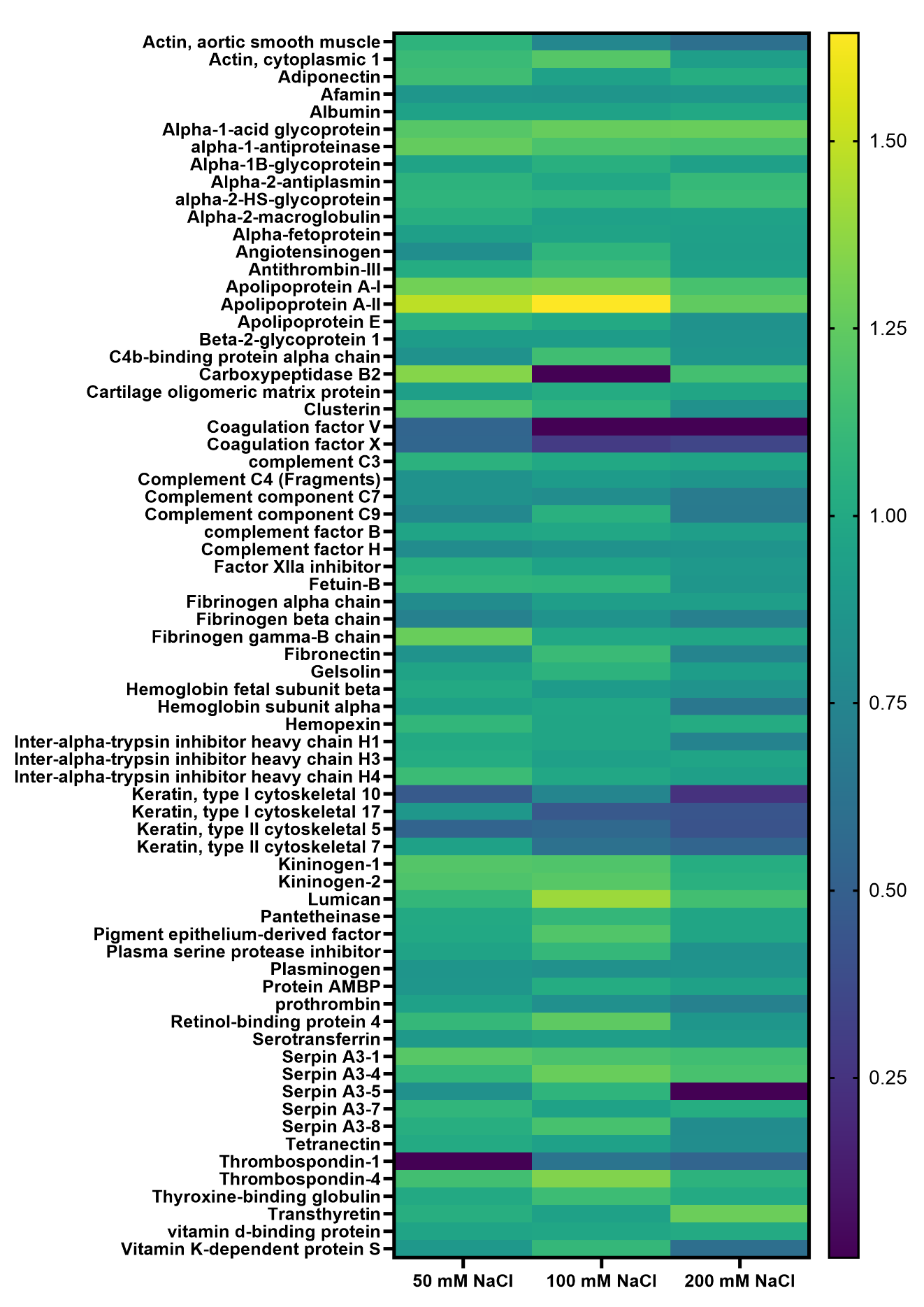

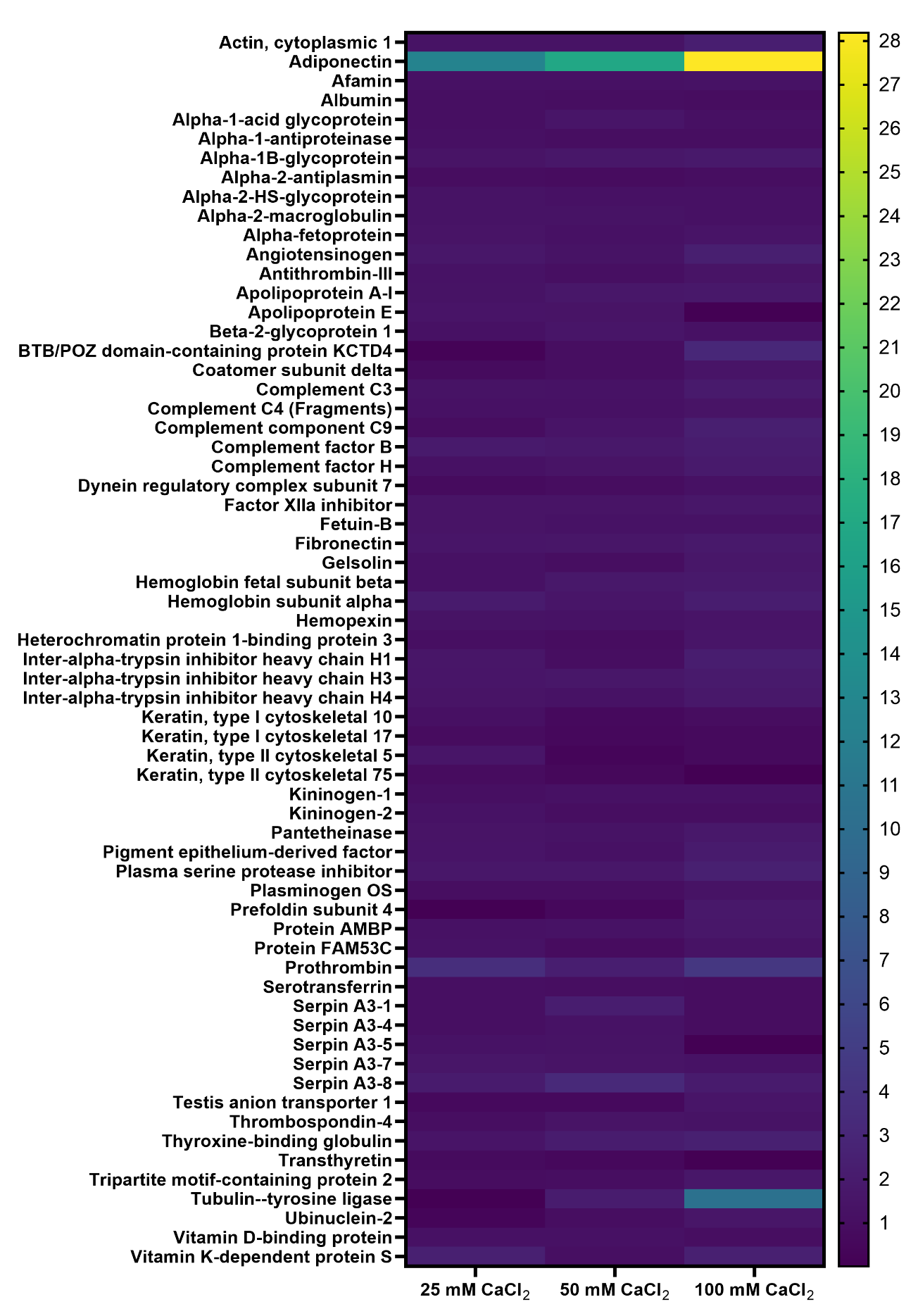


**Figure S3. Heat map of relative protein abundance in the protein corona detected by LC-MS/MS as compared to 0 mM controls after incubation with NaCl**

**Figure S4. Heat map of relative protein abundance in the protein corona detected by LC-MS/MS as compared to 0 mM controls after incubation with CaCl_2_**


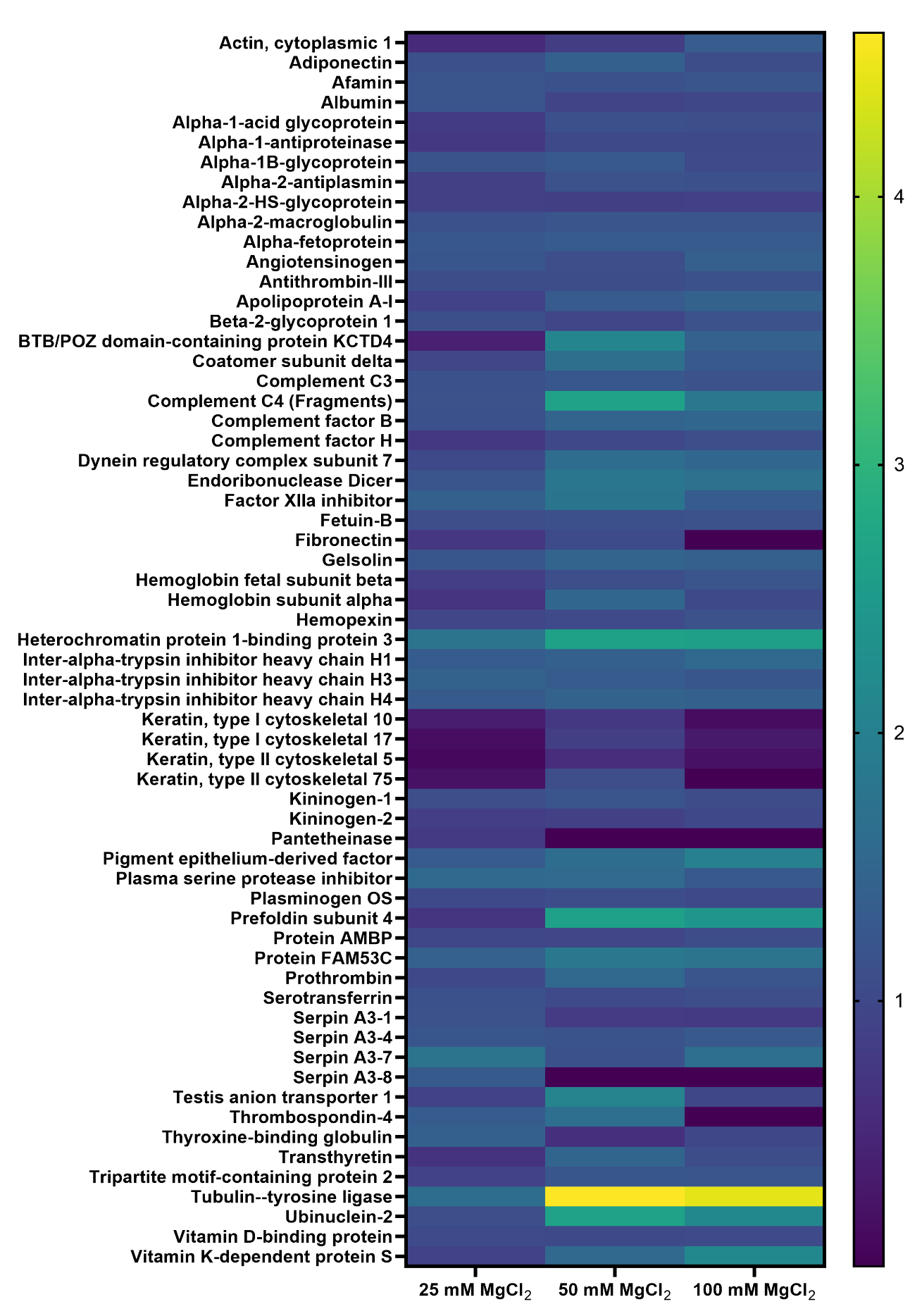


**Figure S5. Heat map of relative protein abundance in the protein corona detected by LC-MS/MS as compared to 0 mM controls after incubation with MgCl_2_**


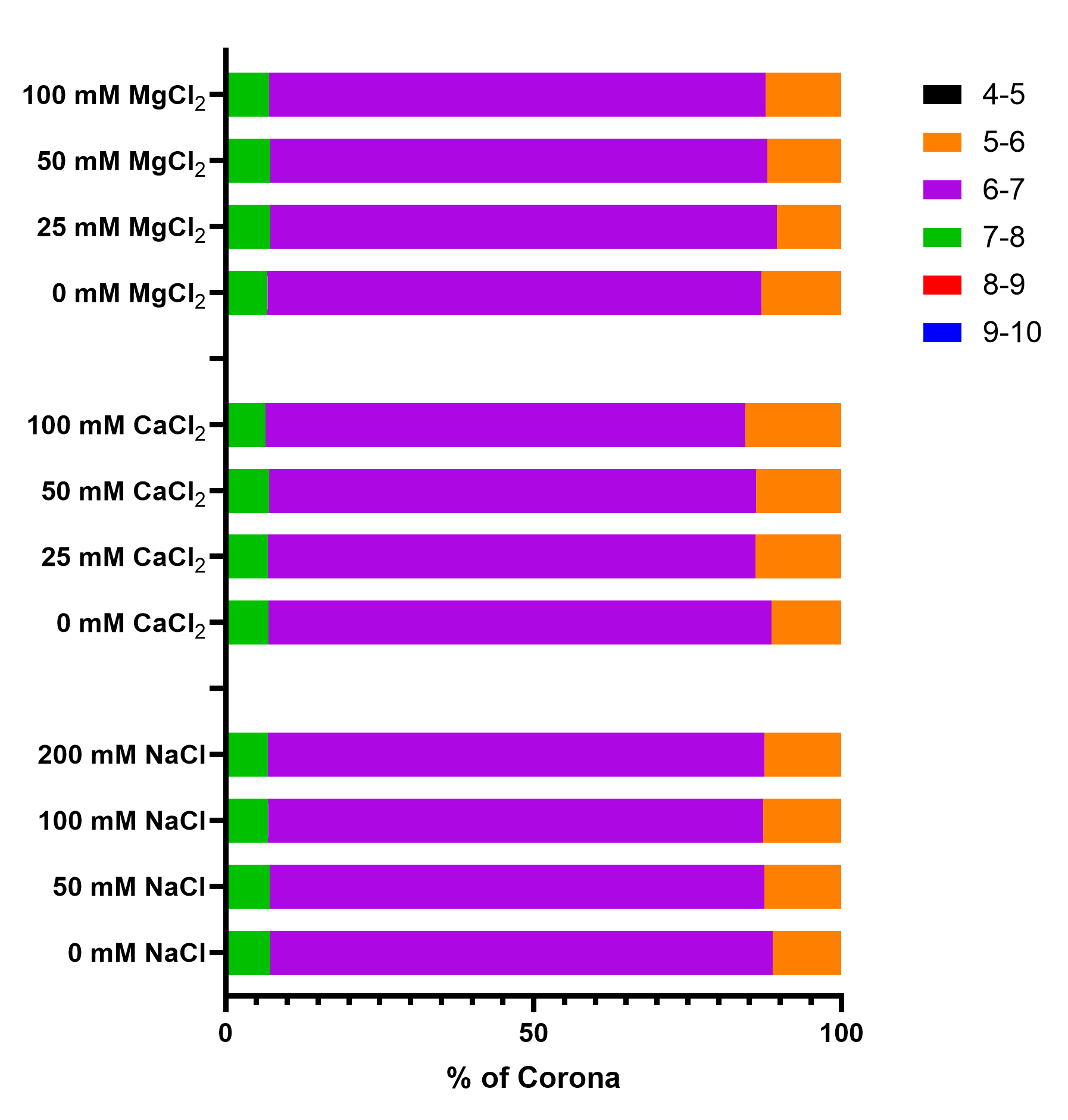


**Figure S6. Protein corona on PEG-b-PLA polymersomes after incubation with chaotropic salts organized by isoelectric point (PI).** No obvious changes are observable due to the large number of proteins with PI between 6 and 7. This includes albumin, which is the most abundant protein in FBS and in human serum.


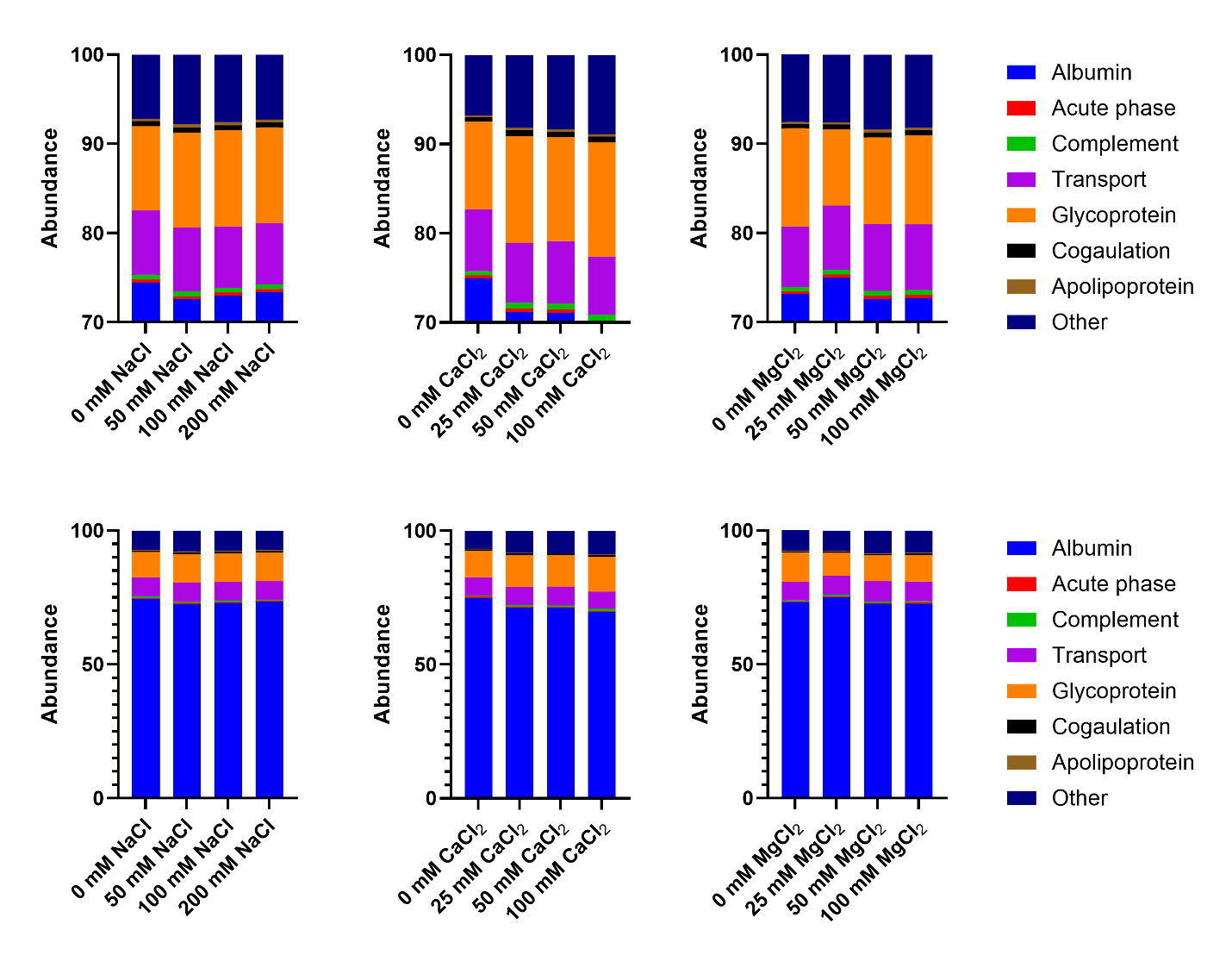


**Figure S7.** **Protein corona characterized by protein function for PEG-b-PLA polymersomes incubated (A) NaCl, (B) CaCl_2_, and (C) MgCl_2_.** This figure includes the entirety of the abundance, including albumin.
